# Sodium Glucose Cotransporter Type 2 Inhibitors Improve Cardiorenal Outcome of Patients With Coronary Artery Disease: A Meta-Analysis

**DOI:** 10.3389/fendo.2022.850836

**Published:** 2022-03-07

**Authors:** Wen Wei, Jin Liu, Shiqun Chen, Xinghao Xu, Dachuan Guo, Yibo He, Zhidong Huang, Bo Wang, Haozhang Huang, Qiang Li, Jiyan Chen, Hong Chen, Ning Tan, Yong Liu

**Affiliations:** ^1^ Department of Cardiology, Guangdong Cardiovascular Institute, Guangdong Provincial People’s Hospital, Guangdong Academy of Medical Sciences, Guangzhou, China; ^2^ Department of Guangdong Provincial Key Laboratory of Coronary Heart Disease Prevention, Guangdong Cardiovascular Institute, Guangdong Provincial People’s Hospital, Guangdong Academy of Medical Sciences, Guangzhou, China; ^3^ Department of Endocrinology, Longyan First Affiliated Hospital of Fujian Medical University, Longyan, China; ^4^ The Second School of Clinical Medicine, Southern Medical University, Guangzhou, China; ^5^ Department of Cardiology, Sun Yat-sen Memorial Hospital, Sun Yat-sen University, Guangzhou, Guangdong, China; ^6^ Department of Endocrinology, Zhujiang Hospital, Southern Medical University, Guangzhou, China; ^7^ Guangdong Provincial People’s Hospital, School of Medicine, South China University of Technology, Guangzhou, China

**Keywords:** sodium glucose cotransporter type 2 inhibitors, improve, coronary artery disease, cardiorenal outcomes, meta-analysis

## Abstract

**Objective:**

Sodium glucose cotransporter type 2 inhibitors (SGLT-2i) are beneficial for cardiorenal outcomes in patients with type 2 diabetes mellitus (T2DM), heart failure (HF) or chronic kidney disease (CKD). However, whether or not the patients with coronary artery disease (CAD) have prognostic benefit from SGLT-2i treatment has not been fully studied. The purpose of this meta−analysis is to determine the prognostic benefit of SGLT-2i administration in CAD patients.

**Methods:**

We searched the PubMed, Embase and Cochrane Library from inception until October 15, 2021. We included randomized controlled trials (RCTs) reporting the effect of SGLT-2i on major adverse cardiovascular event (MACE), hospitalization for heart failure (HHF), cardiovascular (CV) death and cardiorenal parameters in CAD patients. Hazard ratio (HR) with 95% confidence interval (CI) and mean difference (MD) from trials were meta-analyzed using fixed-effects models.

**Results:**

Nine trials enrolling 15,301 patients with CAD were included in the analyses. Overall, SGLT2i were associated with a reduced risk of MACE (HR: 0.84; 95% CI 0.74–0.95; I^2^ = 0%), HHF (HR: 0.69; 95% CI 0.58–0.83; I^2^ = 0%) and a composite of CV death or HHF (HR: 0.78; 95% CI 0.71–0.86; I^2^ = 37%) in CAD patients. Compared with control group, estimated glomerular filtration rate (eGFR) level decreased less in SGLT-2i group (mean difference [MD] = −3.60, 95% CI, −5.90 to −1.30, p = 0.002; I^2^ = 0%).

**Conclusions:**

SGLT-2i can improve cardiorenal outcomes in CAD patients. Further RCTs and real world studies are need to investigate the effect of SGLT2i on CAD patients.

**Systematic Review Registration:**

PROSPERO, CRD42021258237.

## Introduction

Coronary artery disease (CAD) is one of the most common causes of morbidity and mortality worldwide (1, 2). CAD is usually concurrent with type 2 diabetes mellitus (T2DM), chronic kidney disease (CKD) and heart failure (HF), and the risk of cardiovascular (CV) death increases significantly with increasing comorbidities (3–6).

Sodium glucose cotransporter type 2 inhibitors (SGLT-2i) are new glucose-lowering drugs, and have been approved globally for the treatment of T2DM, either as monotherapy or in combination with other hypoglycemic drugs (7). A number of randomized controlled trials (RCTs) have shown that SGLT-2i have benefits on reducing major adverse cardiovascular event (MACE), hospitalization for heart failure (HHF) and progression of renal insufficiency in patients with T2DM (8–11). Among patients with CKD or HF, regardless of the presence or absence of diabetes, the risk of worsening cardiorenal function or CV death was lower among those who received SGLT2i than placebo treatment (12–14). SGLT-2i also reduced the burden of cardiovascular complications and all-cause admission to hospital in T2DM with atherosclerotic cardiovascular disease (ASCVD) (15). However, the cardiorenal benefit of SGLT2i in patients with high-risk and prevalent CAD is uncertain.

Therefore, we conducted a meta-analysis to determine the cardiorenal benefit of SGLT2i in patients with CAD.

## Materials and Methods

### Protocol and Guidance

The meta-analysis was registered in PROSPERO (CRD42021258237) and performed based on the preferred reporting items for systematic review and meta-analyses (PRISMA) guidelines (16).

### Search Strategy

We searched the PubMed, Embase and Cochrane Library from inception until October 15, 2021, with English language restrictions. Search keywords included “coronary artery disease”, “myocardial infarction”, “heart failure”, “type 2 diabetes”, “+”, “Sodium Glucose Transporter 2 Inhibitors”, “SGLT2”, “+”, “randomized controlled trial”, “clinical trial”, “trial”, “secondary”. The medical subject headings (MeSH) or title/abstract limiting searching were used when the searching databases with option was available. The search strategies are detailed in **Table S1**. In addition, the search was restricted to human studies with animal studies excluded. Two investigators independently searched for papers, screened titles and abstracts of the retrieved articles, reviewed the full-texts, and selected the articles for inclusion.

### Study Selection

Eligible patients included CAD with or without T2DM. Patients in the intervention group were treated with SGLT2i (Canagliflozin, Dapagliflozin, Empagliflozin, Ertugliflozin, Sotagliflozin, Luseogliflozin, Ipragliflozin, Tofogliflozin), while the control group consisted of placebo or other hypoglycemic drugs. The primary outcome was MACE, defined as a composite of CV death, nonfatal myocardial infarction, or nonfatal stroke. Other 5 secondary outcomes included CV death, HHF, a composite of CV death or HHF, and changes of N-terminal prohormone of brain natriuretic peptide (NT-proBNP) and estimated glomerular filtration rate (eGFR) levels.

### Data Extraction and Quality Assessment

Key information was extracted from nine RCTs, namely, study name or first author name, publication year, study design, mean follow-up, number of participants and characteristics, subgroup analysis types, drug of intervention and control group, the primary outcomes and secondary outcomes.

The quality of RCT was assessed with the Cochrane risk bias tool (17), which contained five domains: random sequence generation (selection bias), allocation concealment (selection bias), blinding (performance bias and detection bias), incomplete outcome data (attrition bias), and selective reporting (reporting bias).

All the data extraction and quality assessment were done independently by two investigators, and the divergence was settled by discussion and consensus or by third-party arbitration.

### Statistical Analysis

Meta-analyses were conducted using RevMan (version 5.3; Copenhagen: The Nordic Cochrane Centre, The Cochrane Collaboration, 2014) and R programming language, version 4.0.3 (R Foundation for Statistical Computing, Vienna, Austria). We used the Hazard Ratio (HR) with 95% confidence interval (CI) to evaluate clinical outcomes (MACE, CV death and HHF) in patients with CAD receiving standard treatment with or without SGLT-2i. Similarity, the mean difference (MD) was calculated to evaluate changes of NT-proBNP and eGFR levels in patients with CAD receiving standard treatment with or without SGLT-2i in 6-month treatment. I^2^ statistic was used to assess heterogeneity. I^2^ ≤50% was considered to represent low heterogeneity, and higher than 50% was regarded as high. A fixed-effect model was used to pool the results when I^2^ ≤50%, while a random-effect model was used when I^2^ >50% (18).

## Results

### Search Results

**Figure 1:**
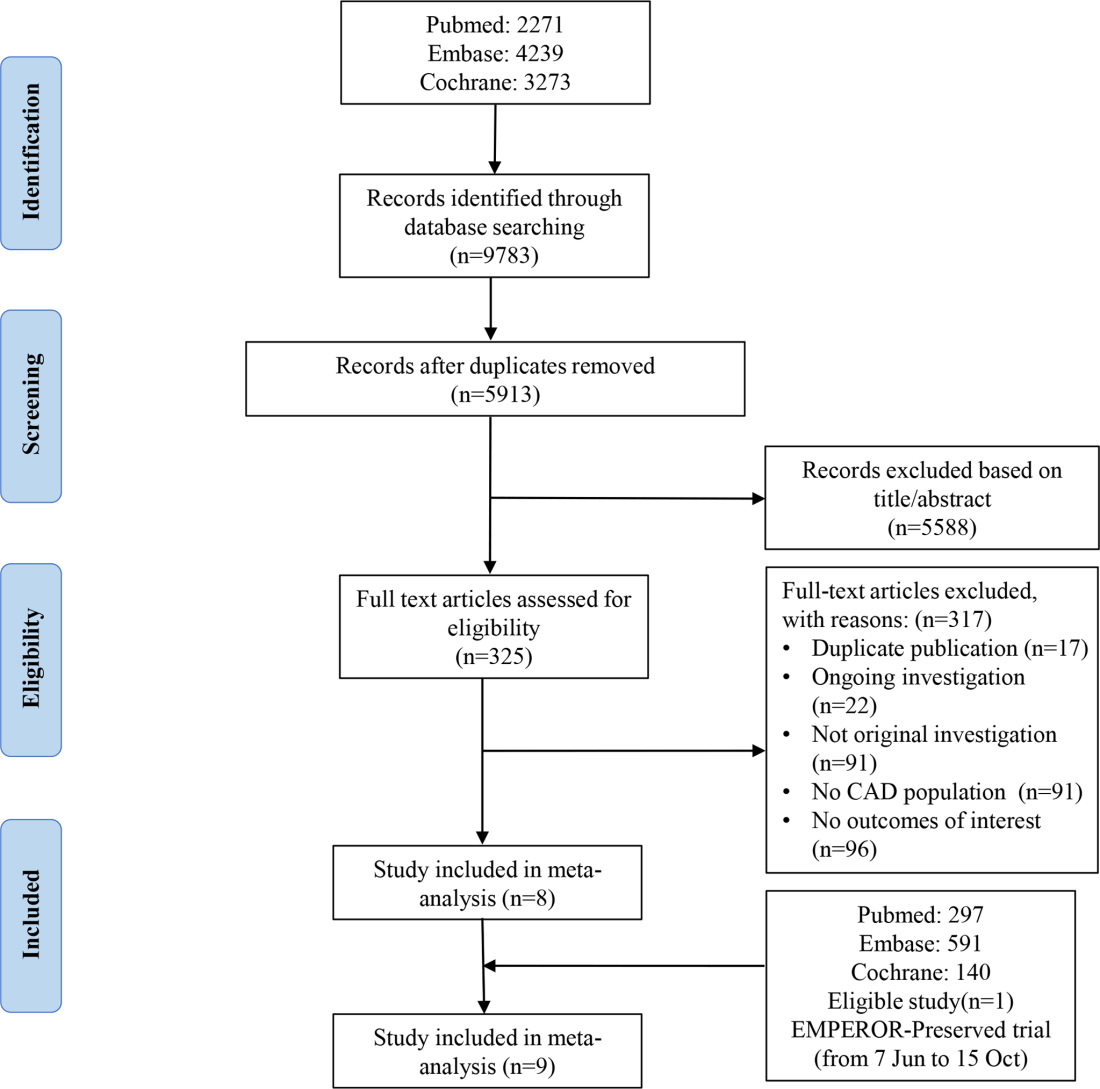
Eligibility of the studies for inclusion in the meta-analysis.

Until June 6, 2021, 9,783 articles were retrieved from PubMed, Embase and Cochrane Library. After removing duplicate studies, the remaining 5,913 articles were screened. After reading 325 eligible full-text articles, 317 were excluded, and 8 studies met our inclusion criteria (8, 13, 19–24). After completing the manuscript, we searched the newly published literatures in PubMed, Embase and Cochrane Library. From June 7 to October 15, 1028 articles were retrieved. After removing duplicate studies, screening title and abstract, and reading the eligible full-text articles, the remaining 1 study met our inclusion criteria (25). In total, 9 studies were included in meta-analysis. The systematic search results are presented in **Figure 1**.

### Study Characteristics

A total of 9 studies of SGLT-2i enrolling 15,301 patients with CAD were included in final analysis (1 main-analysis and 8 sub-analyses of published RCT trials, **Table 1**). Among them, 5 trials concerned empagliflozin, 3 trials involved dapaglifozin and 1 trial discussed sotagliflozin. In addition, 8 studies reported comparison with placebo, and 1 study reported comparison with dipeptidyl peptidase-4 inhibitor (DPP4i): vildagliptin. The detailed baseline characteristics of each study are presented in **Tables 1** and **2**.

**Table 1:** Summary of the characteristics of the included studies.

Source	Study year	Mean Follow-up	Population	Sample size	Subgroup	Treatment	Outcome: MACE		Outcome: HHF		Outcome: CV death		Outcome: Composite of CV death or HHF	
							Events/Patients, n/N		Events/Patients, n/N		Events/Patients, n/N		Events/Patients, n/N	
							Treatment	Placebo	Treatment	Placebo	Treatment	Placebo	Treatment	Placebo
EMPA-REG OUTCOME 2015	2015	3.1 years	Type 2 diabetes and high CV risk	7,020	CAD prespecified	Empagliflozin	261/2,732	152/1,340	NR	NR	90/2,732	63/1,340	NR	NR
Substudy of DECLARE-TIMI 58 2019	2018	4.2 years	Patients with type 2 diabetes who had or were at risk for atherosclerotic cardiovascular disease	17,160	Previous MI prespecified	Dapaglifozin	270/1,777	321/1,807	81/1,777	114/1,807	87/1,777	96/1,807	153/1,777	190/1,807
Substudy of DAPA-HF 2021	2019	18.2 months	HF with reduced ejection fraction, with and without type 2 diabetes	4,744	Cause of heart failure: ischemic prespecified	Dapaglifozin	NR	NR	118/1,316	173/1,358	152/1,316	170/1,358	222/1,316	284/1,358
EMPEROR-Reduced 2020	2020	16 months	HF with reduced ejection fraction, with and without type 2 diabetes	3,730	Cause of heart failure: ischemic prespecified	Empagliflozin	NR	NR	NR	NR	NR	NR	207/983	236/946
SOLOIST-WHF 2020	2020	9 months	Type 2 diabetes mellitus who were recently hospitalized for worsening heart failure	1,222	Cause of heart failure: ischemic	Sotagliflozin	NR	NR	NR	NR	NR	NR	Total patients: 712	
EMPEROR-Preserved 2021	2021	26.2 months	HF with preserved ejection fraction, with and without type 2 diabetes	5,988	Cause of heart failure: ischemic	Empagliflozin	NR	NR	NR	NR	NR	NR	157/1,079	177/1,038

MACE, major adverse cardiovascular event (Composite of CV death/nonfatal MI/nonfatal stroke); CV, cardiovascular; HF, heart failure; HHF, hospitalization for heart failure; MI, myocardial infarction; CAD, coronary artery disease; NR, not reported.

**Table 2:** Summary of the characteristics of the included studies.

Source	Study period	Design	Mean Follow-up	Population	Treatment group	Control group	Primary outcome	Outcome: NT-proBNP	Outcome: eGFR
								Treatment/control	Treatment/control
Arintaya Phrommintikul (22)	NR	Prospective randomised double-blinded study	6 months	T2DM with CAD	Dapagliflozin	vildagliptin	Cardiometabolic parameters and inflammatory biomarkers	21/22	21/22
Tamique Mason (23)	Nov 2016 to Apr 2018	Prespecified substudy of EMPA-HEART CardioLink-6 Trial	6 months	T2DM with CAD	Empagliflozin	placebo	Changes in left ventricular ECV, LVMi, iICV, iECV and the fibrosis biomarkers sST2 and MMP-2	NR	39/35
Kosuke Mozawa (24)	Feb 2018 to Mar 2019	Substudy of the Embody Trial	24 weeks	T2DM with AMI	Empagliflozin	placebo	Changes in renal functional markers	32/33	46/50

CAD, coronary artery disease; AMI, acute myocardial infarction; NT-proBNP, N-terminal prohormone of brain natriuretic peptide; eGFR, estimated glomerular filtration rate; NR, not reported; ECV, extracellular volume; LVMi, left ventricular mass index; iICV, indexed intracellular compartment volume; iECV, indexed extracellular compartment volume; sST2, soluble suppressor of tumorgenicity; MMP, matrix metalloproteinase.

### Risk of Bias Within Studies

Egger’s test and funnel plots were not applied for publication bias assessment because less than 10 studies were included. Quality evaluation was conducted using the Cochrane systematic evaluation method. Most studies had a low risk of overall bias, as shown in **Supplementary Figure 1**.

### Difference in Cardiovascular Events

**Figure 2:**
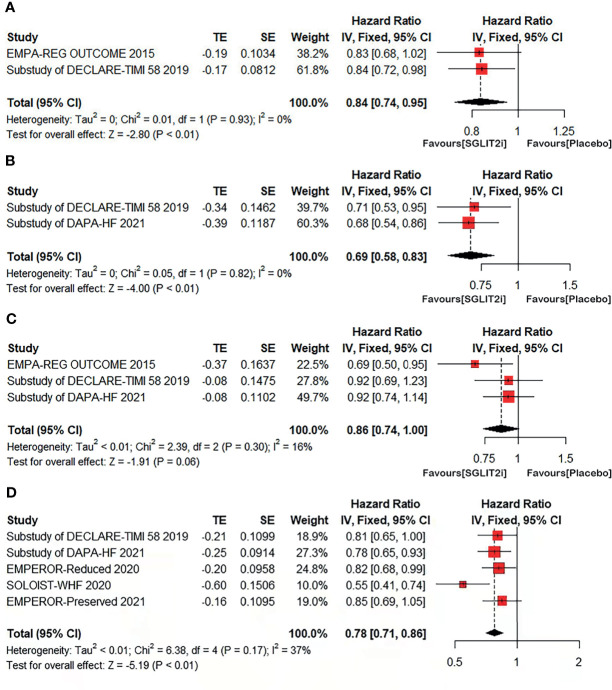
Forest plot of meta-analysis for the comparison of cardiovascular outcomes between the SGLT-2i and placebo groups. **(A)** MACE: major adverse cardiovascular event, **(B)** HHF: hospitalization for heart failure, **(C)** CV death: cardiovascular death, **(D)** a composite of CV death or HHF.

For the primary outcome, two articles contained MACE outcome, with a total of 7,656 patients (4,509 in SGLT-2i group and 3,147 in placebo group). In SGLT-2i group, 531 (11.8%) patients met MACE outcome, while 473 (15.0%) patients in placebo group. Meta-analysis result showed that the risk of MACE was significantly lower in patients using SGLT-2i than in placebo group (HR: 0.84; 95% CI 0.74–0.95; P <0.01; I^2^ = 0 =%; **Figures 2A**, **4**).

Two articles contained heart failure hospitalization (HHF) outcome, with a total of 6,258 patients (3,093 in SGLT-2i group and 3,165 in placebo group). In SGLT-2i group, 199 (6.4%) patients met HHF outcome, while 287 (9.1%) patients in placebo group. Meta-analysis result showed that the risk of HHF was significantly lower in patients using SGLT-2i than in placebo group (HR: 0.69; 95% CI 0.58–0.83; P <0.01; I^2^ = 0%; **Figures 2B**, **4**).

Cardiovascular death (CV death) outcome were included in three articles involving a total of 10,330 patients (5,825 in SGLT-2i group and 4,505 in placebo group). In SGLT-2i group, 329 (5.6%) patients met CV death outcome, while 329 (7.3%) patients in placebo group. The result showed that no significant difference in the risk of CV death was found between the two groups (HR: 0.86; 95% CI 0.74–1.00; P = 0.06; I^2^ = 16%; **Figures 2C**, **4**).

Five articles included CV death/HHF outcome for a total of 11,016 patients. Meta-analysis result showed that the risk of CV death/HHF was significantly lower in patients using SGLT-2i than in placebo group (HR: 0.78; 95% CI 0.71–0.86; P <0.01; I^2^ = 37%; **Figure 2D**, **4**).

### Difference in Mean Change in Cardio-Renal Parameters

**Figure 3:**
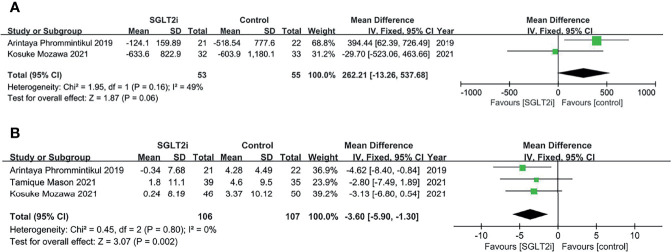
Forest plot of meta-analysis for the comparison of cardio-renal parameters between the SGLT-2i and control groups. **(A)** decrease of NT-proBNP levels, **(B)** decrease of eGFR levels.

We used NT-proBNP to represent cardiac function. Two articles included change of NT-proBNP in 6-month treatment, with a total of 108 patients (53 in the SGLT-2i group and 55 in the control group). Meta-analysis results showed the decrease of NT-proBNP in control group was more than that in SGLT2i group but without significant difference between the two groups (MD = 262.21, 95% CI, −13.26 to 537.68, p = 0.06; I^2^ = 49%; **Figure 3A**).

Renal function was assessed by eGFR. Three articles contained change of eGFR in 6-month treatment, with a total of 213 patients (106 in the SGLT-2i group and 107 in the control group). The pooled effect size showed less decrease of eGFR levels (MD = −3.60, 95% CI, −5.90 to −1.30, p = 0.002; I^2^ = 0%) in SGLT-2i group compared to control group; **Figure 3B**).

## Discussion

To the best of our knowledge, this is the first study to compare SGLT-2i treatment with placebo or other glucose-lowering treatment in comprehensive outcomes of cardiovascular events and cardiorenal parameters in patients with CAD. The main findings of this meta-analysis demonstrated that in patients with CAD, SGLT-2i significantly reduced cardiovascular events compared with the placebo. Additionally, we found SGLT-2i had a protective effect on renal function decline in these patients.

Recently, several meta-analyses have demonstrated that SGLT-2i could improve cardiorenal outcomes in T2DM patients with cardiovascular diseases (26–30). A meta-analysis about a subpopulation of subjects with CAD showed a 15% risk reduction (relative risk [RR] 0.85) for MACE outcome and a 39% risk reduction (RR 0.61) for HHF outcome. However, no benefit was shown for CV death (31). Consistent with above study, our study showed a 16% risk reduction for MACE outcome and a 32% risk reduction for HHF outcome evaluated by HR with 95% CI. Similarly, no benefit was observed for SGLT-2i in CV outcomes. This should be further explored in future RCTs and real-world research on CAD population. In addition, we also analyzed a composite of CV death or HHF outcome, showing that SGLT-2i significantly reduces a 23% risk of CV death or HHF. The risk reduction was mostly attributed to the decreased risk of HHF. Our results reaffirm the indication of SGLT-2i for subjects with CAD, especially in patients with T2DM or heart failure.

SLGT-2i has been shown to reduce the risk of dialysis, transplantation, or death due to kidney disease in individuals with T2DM (32). A meta-analysis also reported that SGLT-2i reduced the risk of acute kidney injury (AKI) with or without hospitalization in randomized trials and the real-world setting (33). Our study showed that the decrease of eGFR in SGLT-2i group was less than that in control group, and eGFR was greater than 60 ml/min/1.73 m^2^ in both groups at the end of the study (period: 6 months), which also suggested that SGLT-2i had a protective effect on renal function decline. We also found that after a 6-month treatment, the decrease in NT-proBNP was more in control than SGLT2i for unknown reasons driven by that one study with vildagliptin as the control group. However, there was no significant difference between the two groups. The reasons may also be that the number of studies we included was small and the intervention of the control group was different in the two studies. Therefore, we will explore this issue further in future studies.

**Figure 4:**
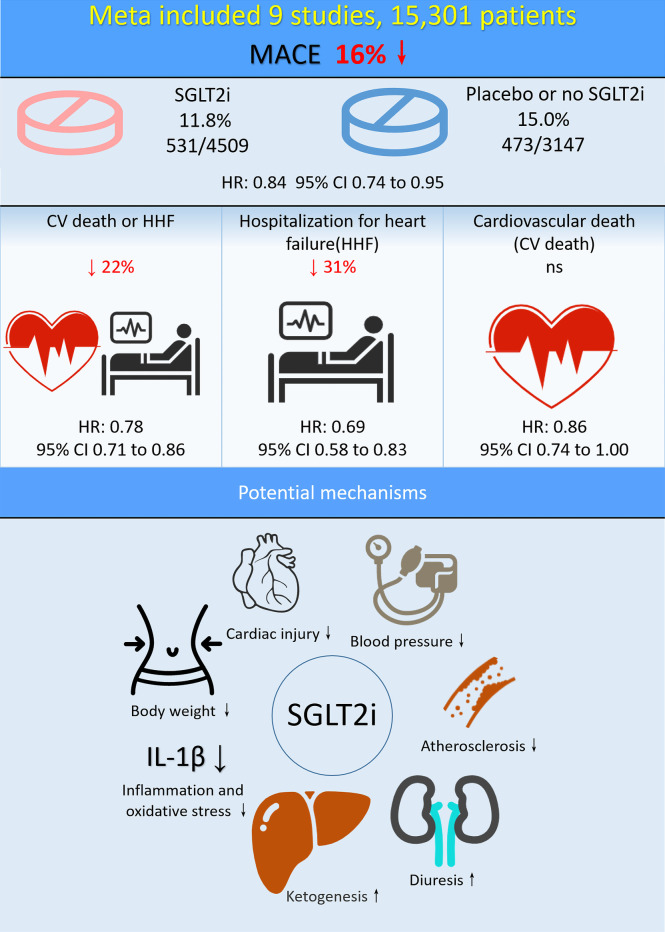
Central illustration: Meta-analysis and potential mechanisms of SGLT-2i in CAD patients.

Among patients with CAD, those with diabetes comprise a higher-risk subgroup (34). In these patients, the best possible glycemic control obtained with the older glucose-lowering medications (different combinations of metformin, sulfonylureas, thiazolidinediones, glinides, and insulin) is unlikely to improve their cardiovascular outlook (35). The newer glucose-lowering drugs, in particular SGLT-2i, have demonstrated a consistent effect of reducing the risk of cardiovascular events in both controlled trials and real-world research. Possible mechanisms for the cardiovascular benefits of SGLT-2i include diuresis, lowering blood pressure and body weight, improving atherosclerosis, and reducing inflammation and oxidative stress (36). Another proposed mechanism is inhibition of sodium hydrogen exchanger 3 (NHE3) in the kidney and heart. In the kidney, this may increase sensitivity to diuretics and natriuretic peptides, leading to decreased preload in heart failure patients. In cardiac tissue, inhibition of NHE3 could limit cardiac injury, systolic dysfunction and remodeling (27, 37). Recent studies suggest that the increased level of ketone bodies related to SGLT-2i use might mediate part of the beneficial effect (38, 39) (**Figure 4**).

Acute myocardial infarction (AMI) is the most serious manifestation of CAD, leading to significant mortality and disease burden (40). AMI patients who had a history of stroke, combined with hypertension and diabetes mellitus had a relatively higher mortality, considering related to the factors as combined with more disease, relatively complex and worse vascular condition. SGLT2i are a unique class of medications that not only improve cardiorenal outcomes, but also have a demonstrated impact on reducing cardiovascular risk factors such as hyperglycemia, hypertension, dyslipidemia and excessive weight (41). The results of the China Heart Failure Registry Study (China-HF) showed that CAD had become the main causes of HF in China (42). Results of our meta-analysis showed that SGLT-2i could reduce MACE and HHF in patients with CAD, again confirming the indication of SGLT2i in patients with CAD, especially in high risk patients with hypertension, T2DM or HF. Rational use of SGLT2i in CAD patients can reduce the mortality. As for CAD patients without T2DM or HF, further studies are needed to demonstrate the benefits of SGLT-2i. Moreover, there was no clear evidence of additional safety concerns over SGLT-2i use in the treatment of cardiovascular disease. Fear of causing AKI should not stop practitioners prescribing SGLT-2i.

### Limitations

Our study had some limitations. First, the number of included trials included was relatively small, which may prevent robust assessment of outcomes. Further validation is needed in future RCT studies. Second, the CAD patients included in our meta-analysis had either diabetes or heart failure, and the cardiorenal benefits of SGLT2i in DM or HF patients were well established. As for CAD patients without DM or HF, further studies are needed to demonstrate the benefits of SGLT-2i. Third, in trials where decrease of NT-proBNP was used as an outcome, control interventions were different, which may influence the assessment of outcomes. Finally, the meta-analysis did not use patient level data.

### Conclusion

In conclusion, SGLT-2i treatment can reduce the risk of major cardiovascular events and have a protective effect on renal function decline in CAD patients with T2DM or HF. Prospective studies exploring the effect of SGLT-2i on cardiovascular endpoint improvement in general CAD patients should be carried out in the future.

## Data Availability Statement

The original contributions presented in the study are included in the article/**Supplementary Material**. Further inquiries can be directed to the corresponding authors.

## Author Contributions

Substantial contributions to the conception and design of the meta−analysis (YL, NT, HC). Data collection (WW, XHX, DCG, ZDH, BW, HZH, QL). Data analysis and/or interpretation of data for the work (WW, SQC, YBH, JYC). Drafting of the work or revising it critically for important intellectual content (WW, JL, XHX, DCG). All authors listed have made a substantial, direct, and intellectual contribution to the work and approved it for publication.

## Funding

This research was funded and supported by the Guangdong Provincial Science and Technology Plan Project (2017B030314041), the Guangdong Provincial Fund for Clinical Medications (2019ZH01), the Multi-center study on key techniques for prevention, diagnosis and treatment of high risk coronary artery disease (DFJH2020026), the Study on the function and mechanism of the potential target for early warning of cardiorenal syndrome after acute myocardial infarction based on transmoomics (DFJH201919), and the Natural Science Foundation of Guangdong Province General Project (2020A1515010940).

## Conflict of Interest

The authors declare that the research was conducted in the absence of any commercial or financial relationships that could be construed as a potential conflict of interest.

## Publisher’s Note

All claims expressed in this article are solely those of the authors and do not necessarily represent those of their affiliated organizations, or those of the publisher, the editors and the reviewers. Any product that may be evaluated in this article, or claim that may be made by its manufacturer, is not guaranteed or endorsed by the publisher.

## Supplementary Material

Supplementary Figure 1 Quality assessment for the evidences.
