# Supplementary figures and images for: Sodium Glucose Cotransporter Type 2 Inhibitors Improve Cardiorenal Outcome of Patients With Coronary Artery Disease: A Meta-Analysis
Source: Front Endocrinol (Lausanne). 2022 Mar 7;13:850836. doi: 10.3389/fendo.2022.850836 (PMC8940298; doi:10.3389/fendo.2022.850836)

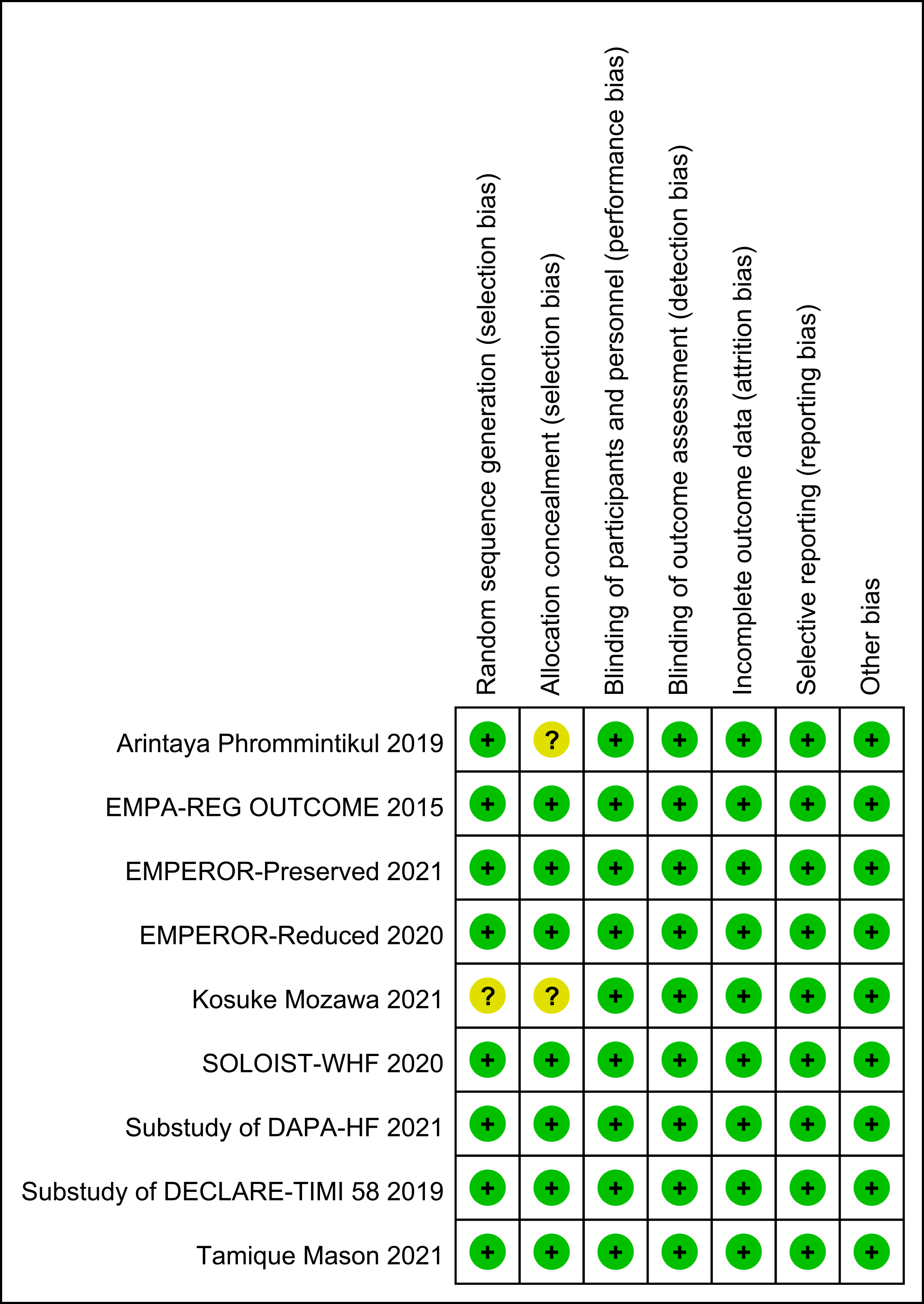

Supplement: Supplementary Figure 1 — Quality assessment for the evidences. [file Image_1.tif]
