# Supplementary material for: Sodium Glucose Cotransporter Type 2 Inhibitors Improve Cardiorenal Outcome of Patients With Coronary Artery Disease: A Meta-Analysis
Source: Front Endocrinol (Lausanne). 2022 Mar 7;13:850836. doi: 10.3389/fendo.2022.850836 (PMC8940298; doi:10.3389/fendo.2022.850836)
Supplement: Supplementary file 2 [file Table_1.docx]

**Supplementary material**

**Table S1. Search strategy for each database.**

| database | Search strategy |
| --- | --- |
| Pubmed | #1 (Sodium-Glucose Transporter 2 Inhibitors[MeSH Terms] OR SGLT2 inhibitor*[Title/Abstract] OR SGLT 2 inhibitor*[Title/Abstract] OR SGLT-2 inhibitor*[Title/Abstract] OR gliflozin*[Title/Abstract] OR Canagliflozin[Title/Abstract] OR Dapagliflozin[Title/Abstract] OR Empagliflozin[Title/Abstract] OR Ertugliflozin[Title/Abstract] OR Sotagliflozin[Title/Abstract] OR Luseogliflozin[Title/Abstract] OR Ipragliflozin[Title/Abstract] OR Tofogliflozin[Title/Abstract] OR "sodium glucose co-transporter type 2 inhibitor*"[Title/Abstract] OR "sodium glucose cotransporter type 2 inhibitor* "[Title/Abstract] OR "sodium glucose transporter type 2 inhibitor*"[Title/Abstract] OR "sodium glucose co-transporter 2 inhibitor*"[Title/Abstract] OR "sodium glucose cotransporter 2 inhibitor*"[Title/Abstract] OR "sodium glucose transporter 2 inhibitor*"[Title/Abstract] OR "sodium dependent glucose co-transporter 2 inhibitor*"[Title/Abstract] OR "sodium dependent glucose cotransporter 2 inhibitor*"[Title/Abstract] OR "sodium dependent glucose transporter 2 inhibitor*"[Title/Abstract] OR "sodium glucose linked cotransporter 2 inhibitor*"[Title/Abstract] OR "sodium glucose linked transporter 2 inhibitor*"[Title/Abstract])  #2 (coronary artery disease[MeSH Terms] OR coronary heart disease*[Title/Abstract] OR coronary artery disease* [Title/Abstract] OR atherosclerotic heart disease* [Title/Abstract] OR myocardial infarction* [Title/Abstract] OR acute coronary syndrome* [Title/Abstract] OR atherosclerotic cardiovascular disease* [Title/Abstract] OR cardiovascular disease* [Title/Abstract] OR cardiovascular risk factor* [Title/Abstract] OR heart failure[Title/Abstract] OR type 2 diabetes[Title/Abstract])  #3 (trial*[Title/Abstract] OR clinical trial*[Title/Abstract] OR randomized controlled trial* [Title/Abstract] OR post-hoc[Title/Abstract] OR secondary[Title/Abstract])  #4 #1 AND #2 AND #3  #5 #4+Filters: Other Animals  #6 #4 NOT #5 |
| Embase | #1. 'sodium glucose cotransporter 2 inhibitor'/exp  #2. 'sglt2 inhibitor*':ab,ti  #3. 'sglt 2 inhibitor*':ab,ti  #4. 'sglt-2 inhibitor*':ab,ti  #5. gliflozin*:ab,ti  #6. canagliflozin:ab,ti  #7. dapagliflozin:ab,ti  #8. empagliflozin:ab,ti  #9. ertugliflozin:ab,ti  #10. sotagliflozin:ab,ti  #11. luseogliflozin:ab,ti  #12. ipragliflozin:ab,ti  #13. tofogliflozin:ab,ti  #14. 'sodium glucose co-transporter type 2 inhibitor*':ab,ti  #15. 'sodium glucose cotransporter type 2 inhibitor*':ab,ti  #16. 'sodium glucose transporter type 2 inhibitor*':ab,ti  #17. 'sodium glucose co-transporter 2 inhibitor*':ab,ti  #18. 'sodium glucose cotransporter 2 inhibitor*':ab,ti  #19. 'sodium glucose transporter 2 inhibitor*':ab,ti  #20. 'sodium dependent glucose co-transporter 2 inhibitor*':ab,ti  #21. 'sodium dependent glucose cotransporter 2 inhibitor*':ab,ti  #22. 'sodium dependent glucose transporter 2 inhibitor*':ab,ti  #23. 'sodium glucose linked cotransporter 2 inhibitor*':ab,ti  #24. 'sodium glucose linked transporter 2 inhibitor*':ab,ti  #25. #1 OR #2 OR #3 OR #4 OR #5 OR #6 OR #7 OR #8 OR #9 OR #10 OR #11 OR #12 OR #13 OR #14 OR #15 OR #16 OR #17 OR #18 OR #19 OR #20 OR #21 OR #22 OR #23 OR #24  #26. 'coronary artery disease'/exp  #27. 'coronary heart disease*':ab,ti  #28. 'coronary artery disease*':ab,ti  #29. 'atherosclerotic heart disease*':ab,ti  #30. 'myocardial infarction*':ab,ti  #31. 'acute coronary syndrome*':ab,ti  #32. 'atherosclerotic cardiovascular disease*':ab,ti  #33. 'cardiovascular disease*':ab,ti  #34. 'cardiovascular risk factor*':ab,ti  #35. 'heart failure':ab,ti  #36. 'type 2 diabetes':ab,ti  #37. #26 OR #27 OR #28 OR #29 OR #30 OR #31 OR #32 OR #33 OR #34 OR #35 OR #36  #38. trial*:ab,ti  #39. 'clinical trial*':ab,ti  #40. 'randomized controlled trial*':ab,ti  #41. 'post hoc':ab,ti  #42. secondary:ab,ti  #43. #38 OR #39 OR #40 OR #41 OR #42  #44. #25 AND #37 AND #43  #45. #44 AND ('animal experiment'/de OR 'animal model'/de OR 'animal tissue'/de)  #46. #44 NOT #45 |
| Cochrane library | #1 MeSH descriptor: [Sodium-Glucose Transporter 2 Inhibitors] explode all trees  #2 (SGLT2 inhibitor*):ti,ab,kw OR (SGLT 2 inhibitor*):ti,ab,kw OR (SGLT-2 inhibitor*):ti,ab,kw OR (gliflozin*):ti,ab,kw OR (Canagliflozin):ti,ab,kw (Word variations have been searched)  #3 (Dapagliflozin):ti,ab,kw OR (Empagliflozin):ti,ab,kw OR (Ertugliflozin):ti,ab,kw OR (Sotagliflozin):ti,ab,kw OR (Luseogliflozin):ti,ab,kw (Word variations have been searched)  #4 (Ipragliflozin):ti,ab,kw OR (Tofogliflozin):ti,ab,kw OR ("sodium glucose co-transporter type 2 inhibitor*"):ti,ab,kw OR ("sodium glucose cotransporter type 2 inhibitor*"):ti,ab,kw OR ("sodium glucose transporter type 2 inhibitor*"):ti,ab,kw (Word variations have been searched)  #5 ("sodium glucose co-transporter 2 inhibitor*"):ti,ab,kw OR ("sodium glucose cotransporter 2 inhibitor*"):ti,ab,kw OR ("sodium glucose transporter 2 inhibitor*"):ti,ab,kw OR ("sodium dependent glucose co-transporter 2 inhibitor*"):ti,ab,kw OR ("sodium dependent glucose cotransporter 2 inhibitor*"):ti,ab,kw (Word variations have been searched)  #6 ("sodium dependent glucose transporter 2 inhibitor*"):ti,ab,kw OR ("sodium glucose linked cotransporter 2 inhibitor*"):ti,ab,kw OR ("sodium glucose linked transporter 2 inhibitor*"):ti,ab,kw (Word variations have been searched)  #7 #1 OR #2 OR #3 OR #4 OR #5 OR #6  #8 MeSH descriptor: [Coronary Artery Disease] explode all trees  #9 (coronary heart disease*):ti,ab,kw OR (coronary artery disease*):ti,ab,kw OR (atherosclerotic heart disease*):ti,ab,kw OR (myocardial infarction*):ti,ab,kw OR (acute coronary syndrome*):ti,ab,kw (Word variations have been searched)  #10 (atherosclerotic cardiovascular disease*):ti,ab,kw OR (cardiovascular disease*):ti,ab,kw OR (cardiovascular risk factor*):ti,ab,kw OR (heart failure):ti,ab,kw OR (type 2 diabetes):ti,ab,kw (Word variations have been searched)  #11 #8 OR #9 OR #10  #12 #7 AND #11 |
